# Low-level laser therapy for the prevention of low salivary flow rate after radiotherapy and chemotherapy in patients with head and neck cancer*

**DOI:** 10.1590/0100-3984.2014.0144

**Published:** 2016

**Authors:** Fernanda Aurora Stabile Gonnelli, Luiz Felipe Palma, Adelmo José Giordani, Aline Lima Silva Deboni, Rodrigo Souza Dias, Roberto Araújo Segreto, Helena Regina Comodo Segreto

**Affiliations:** 1 PhD, Professor at the Faculdades Metropolitanas Unidas (FMU), São Paulo, SP, Brazil.; 2 Masters Student in Clinical Radiology, Department of Diagnostic Imaging, Escola Paulista de Medicina da Universidade Federal de São Paulo (EPM-Unifesp), São Paulo, SP, Brazil.; 3 PhD, Physicist in Charge of the Medical Physics Sector, Department of Clinical and Experimental Oncology, Escola Paulista de Medicina da Universidade Federal de São Paulo (EPM-Unifesp), São Paulo, SP, Brazil.; 4 MSc, Dental Surgeon (private practice), São Paulo, SP, Brazil.; 5 PhD, Interim Head of the Radiotherapy Sector, Department of Clinical and Experimental Oncology, Escola Paulista de Medicina da Universidade Federal de São Paulo (EPM-Unifesp), São Paulo, SP, Brazil.; 6 Tenured Associate Professor in the Department of Clinical and Experimental Oncology, Escola Paulista de Medicina da Universidade Federal de São Paulo (EPM-Unifesp), São Paulo, SP, Brazil.; 7 PhD, Associate Professor in the Department of Clinical and Experimental Oncology, Escola Paulista de Medicina da Universidade Federal de São Paulo (EPM-Unifesp), São Paulo, SP, Brazil.

**Keywords:** Lasers, semiconductor/therapeutic use, Radiotherapy, Head and neck neoplasms/drug therapy, Terapia a laser de baixa potência, Radioterapia, Quimioterapia, Câncer de cabeça e pescoço

## Abstract

**Objective:**

To determine whether low-level laser therapy can prevent salivary hypofunction after radiotherapy and chemotherapy in head and neck cancer patients.

**Materials and Methods:**

We evaluated 23 head and neck cancer patients, of whom 13 received laser therapy and 10 received clinical care only. An InGaAlP laser was used intra-orally (at 660 nm and 40 mW) at a mean dose of 10.0 J/cm^2^ and extra-orally (at 780 nm and 15 mW) at a mean dose of 3.7 J/cm^2^, three times per week, on alternate days. Stimulated and unstimulated sialometry tests were performed before the first radiotherapy and chemotherapy sessions (N0) and at 30 days after the end of treatment (N30).

**Results:**

At N30, the mean salivary flow rates were significantly higher among the laser therapy patients than among the patients who received clinical care only, in the stimulated and unstimulated sialometry tests (*p* = 0.0131 and *p* = 0.0143, respectively).

**Conclusion:**

Low-level laser therapy, administered concomitantly with radiotherapy and chemotherapy, appears to mitigate treatment-induced salivary hypofunction in patients with head and neck cancer.

## Abstract (pt)

**Objetivo:**

Avaliar o impacto do laser de baixa potência na prevenção de hipofluxo salivar em pacientes portadores de câncer de cabeça e pescoço após radioterapia e quimioterapia.

**Materiais e Métodos:**

Treze pacientes receberam laserterapia e dez receberam cuidados clínicos. Utilizou-se um InGaAlP laser diodo para aplicação intraoral (comprimento de onda de 660 nm, 40 mW de potência e dose média de 10 J/cm^2^) e extraoral (comprimento de onda de 780 nm, 15 mW de potência e dose média de 3,7 J/cm^2^), três vezes por semana, em dias alternados. Sialometrias estimulada e não estimulada foram realizadas antes da primeira sessão de radioterapia e quimioterapia (N0) e 30 dias após o final do tratamento (N30).

**Resultados:**

Em N30, os pacientes submetidos à laserterapia apresentaram médias estatisticamente maiores de fluxo salivar estimulado (p = 0,0131) e não estimulado (*p* = 0,0143), em comparação com os pacientes que receberam apenas cuidados clínicos.

**Conclusão:**

A laserterapia de baixa potência realizada concomitantemente a radioterapia e quimioterapia foi capaz de mitigar a hipofunção das glândulas salivares em pacientes portadores de câncer de cabeça e pescoço após o tratamento oncológico.

## INTRODUCTION

Head and neck cancer (HNC) includes a variety of malignant neoplasms with different characteristics. However, in approximately 95% of the cases, the primary histological type observed is squamous cell carcinoma^(1)^. Cancer of the oral cavity is the most representative type of the disease and is considered a public health problem worldwide^(2,3)^. The most recent estimate indicated that approximately 300,000 new cases would occur worldwide in 2012, and, for 2014, the estimated number of new cases in Brazil was approximately 15,000^(4)^.

Radiotherapy is an important therapeutic modality for healing and controlling HNC, because it allows the eradication of the tumor while preserving the function of the normal tissues of the affected region^(5,6)^. It is adopted as the primary treatment in early stages of the disease. However, in more advanced cases, radiotherapy is usually combined with chemotherapy, surgery, or both^(7-9)^. The total radiation dose used in the treatment with curative intent is based on the site and type of tumor, typically 50-70 Gy in conventional radiotherapy models. In most cases, this dose is distributed in fractions of 1.8 to 2.0 Gy/day, five days a week, over a five to seven week period^(10,11)^.

The non-neoplastic cells included in or adjacent to the irradiation fields during radiotherapy also suffer consequences. The extent and intensity of the cytotoxic effects are determined by treatment factors such as total radiation dose, dose per fraction, volume of the radiation field, dose distribution in tissue volume, the use of chemotherapy, and individual patient characteristics^(12)^. The cytotoxic effects can occur during or shortly after radiotherapy but can also occur months or years after treatment, being referred to, respectively, as acute and late effects^(2,5,13)^.

With regard to chemotherapy, derivatives of platinum and 5-fluorouracil are used primarily in weekly protocols aimed at radiosensitizing the tumor^(14)^. In general, the desired effects of platinum derivatives are due to the interaction with purine bases of DNA, which in turn directly affects the cell replication process. Cisplatin, in particular, binds to the nitrogenous guanine base, inhibiting mitotic activity. These cytotoxic effects are systemic, occurring in tumor and in normal cells as well^(15,16)^. However, many of the mechanisms involved are still unclear, as are the clinical manifestations of cytotoxicity in the various human organs, tissues, and cells^(10)^.

Decreased salivary flow is an extremely common complication in patients with HNC undergoing radiotherapy and chemotherapy. However, the mechanisms by which the glandular function in humans is affected have yet to be well defined^(10,17)^. The onset of decreased salivary flow rate is observed early (in the first days of treatment), becoming more evident after a total dose of 20 Gy has been delivered, which corresponds approximately to the second week of radiotherapy^(18)^.

It is believed that up to 72% of the saliva production present before radiotherapy is recovered after its completion. However, it has been reported that total doses higher than 60 Gy can promote irreversible damage to the salivary glands^(18-20)^. In addition, decreased salivary flow rate is accompanied by changes in the characteristics of the saliva, such as pH, protein concentration, ion concentration, viscosity, and color, which can have a number of deleterious side effects on oral tissues and their basic functions^(19,21,22)^.

There is as yet no fully effective treatment for low salivary flow induced by radiotherapy and chemotherapy^(23)^. Various methods and techniques have been described in the literature in attempts to minimize that side effect, as well as its consequent complications. However, many are palliative and treat only the symptoms^(3)^. The use of artificial saliva, mechanical stimulation, and gustatory stimulation are often not well accepted by patients, and systemic sialagogues, such as pilocarpine and bethanechol, can have significant side effects. Therefore, other solutions are gaining prominence and clinical interest. Among such solutions, we highlight the surgical transposition of major salivary glands, as well as the use of cytoprotectors^(11,24)^, acupuncture^(21)^, and low-level laser therapy^(11,25)^.

Low-level laser therapy has proven effective in the treatment of various conditions or diseases, by promoting biomodulation of the cellular metabolism, as well as because it has analgesic and anti-inflammatory properties without mutagenic and photothermal effects. The conversion of laser energy into useful energy for the cells, due to photochemical and photophysical reactions, can stimulate mitochondrial adenosine triphosphate production, cell proliferation, and protein synthesis^(3,20,26)^. These mechanisms allow the use of low-level laser therapy as a stimulating agent of salivary flow rate in patients with various conditions or diseases involving their reduction, such as Sjögren's syndrome^(20)^, aplasia of salivary glands, use of medications and even patients submitted to radiotherapy and chemotherapy^(25)^.

Because it is noninvasive, affordable and easily applied, low-level laser therapy is available in the clinical routine of most cancer clinics, having long been used for the prevention and treatment of mucositis induced by radiotherapy and chemotherapy^(27)^. However, there is still no standardization of the protocols to be adopted specifically for each condition, which makes clinical dosimetry difficult.

Because of the importance of radiotherapy- and chemotherapy-induced effects on the quality of life of patients with HNC, this study aimed to determine the effectiveness of low-level laser therapy, performed concurrent with radiotherapy and chemotherapy, in the prevention of low salivary flow after the completion of cancer treatment.

## MATERIALS AND METHODS

### Patients

This prospective study was conducted in the Radiotherapy Sector of the Universidade Federal de São Paulo (Unifesp), from June 2010 to August 2012. It included 30 patients with HNC (oral cavity, pharynx, larynx, or occult primary tumor) submitted to conventional 3D radiotherapy, with irradiation fields necessarily encompassing all major salivary glands. The total dose ranged from 66 to 70 Gy, given in fractions of 2 Gy/day, in weekly combination with cisplatin (40 mg/m^2^), accompanied or not by surgery. The radiotherapy was performed with a 6 MV linear accelerator (Varian) or with a ^60^Co teletherapy unit (Alcyon II; CGR MeV), in cervicofacial areas and supraclavicular fossa. All patients were over 18 years of age and had a Karnofsky index ≥ 70.

Patients with diabetes mellitus, autoimmune diseases, infectious diseases, or collagen diseases were excluded, as were those with incipient tumors (stage T1 or T2) limited to the larynx, as well as those with trismus (reduced mouth opening capacity) due to surgical sequelae.

This study was approved by the Unifesp Research Ethics Committee (Ruling no. 0844/10). All research subjects gave written informed consent.

### Clinical procedures

All patients underwent pre-radiotherapy preparation and optimization of the oral cavity-including periodontal and restorative treatment; tooth extraction(s); and removal of factors that could influence the severity of the acute and late effects of radiotherapy (poorly fitting dentures, inadequate restorations, etc.)-and were instructed to discontinue the use of removable prosthetic devices. They were also informed of the most common oral complications and were counseled regarding oral hygiene. In addition, they received clinical treatment involving the prescription of rinses with chamomile tea (five times a day), sodium bicarbonate solution (three times a day), antifungal agents (when necessary), and (for patients with teeth) the daily application of 2% neutral fluoride gel. Every patient was evaluated three times a week during radiotherapy and chemotherapy.

After the patients who dropped out, did not submit to all of the proposed procedures, or died were excluded, only 23 patients could be effectively evaluated. Of those 23 patients, 13 were allocated to receive laser therapy (laser group) and 10 were allocated to receive only conventional medical treatment (control group). There were 8 patients who had previously undergone surgery, and those patients were evenly distributed numerically between the two groups. However, they were allocated randomly, as were all of the other research subjects.

### Laser therapy

Laser therapy was performed with an InGaAlP laser (Twin Laser; MMOptics^®^ Ltd., São Carlos, SP, Brazil), three times a week, on alternate days and always by the same dental surgeon. Laser therapy was initiated before the first radiotherapy/chemotherapy session and ended after the last session, totaling 21 sessions.

The laser was used intraorally at 660 nm and 40 mW, at a mean dose of 10.0 J/cm^2^. The laser irradiation time was 10 seconds per point, according to the emitter tip size (0.04 cm^2^). Always excluding the tumor area, we illuminated three points on each buccal mucous membrane (right and left), three points on the upper labial mucosa and three points on the lower labial mucosa, two points on the hard palate, one point on the soft palate, one point on the dorsum of the tongue, two points on each tongue edge (right and left), one point on each tonsillar pillar membrane (right and left), and two points on the mouth floor.

Also, the laser was used extraorally at 780 nm and 15 mW, at a mean dose of 3.8 J/cm^2^. The laser irradiation time was 10 seconds per point, according to the emitter tip size (0.04 cm^2^). Six points were illuminated in each parotid gland, and two were illuminated in each submandibular gland.

The optical fiber of the laser handpiece was always placed perpendicular to and in contact with the tissue during application. Chemical disinfection (with 70% alcohol) was used in order to clean the appliance and the individual plastic barriers. During treatment, the laser operator and the patient used protective goggles with special lenses.

### Saliva collection

For the assessment of salivary flow rate, unstimulated and stimulated sialometry tests were performed at the first radiotherapy/chemotherapy session, designated time point zero (N0), and at 30 days after the end of treatment (N30). Both tests were performed in accordance with Radiation Therapy Oncology Group (RTOG) 9709-Protocol^(28)^.

In the unstimulated sialometry test, patients were instructed to remain seated, with their eyes open and their head tilted slightly forward, and to keep their face and mouth as still as possible. They were subsequently instructed to swallow all the saliva in their mouth and then allow saliva to accumulate saliva in the floor of their mouth for 60 seconds without swallowing. They were then instructed to expectorate the accumulated volume into a graded collection tube. The procedure was performed four more times, totaling five minutes. The flask with the collected saliva was closed and allowed to rest overnight. In the end, the salivary flow rate per minute, in milliliters (mL), was calculated by determining the arithmetic mean.

In the stimulated sialometry test, patient were first instructed to empty their mouth of any saliva or mucus. A 2% sodium citrate solution was then applied along the side edges of the tongue with the aid of a cotton swab, five times over a two-minute period (at 0, 30, 60, 90, and 120 seconds). As in the unstimulated sialometry test, the flask with the collected saliva was closed and allowed to rest overnight, after which the salivary flow rate per minute, in milliliters (mL), was calculated by determining the arithmetic mean.

### Statistical analysis

For the analysis of categorical variables (gender, ethnicity, alcohol consumption, smoking, primary site, histological type, stage, surgery, and total radiation dose), descriptive statistics were used.

Mean stimulated and unstimulated salivary flow rates were submitted to analytical statistical analysis. The Wilcoxon test was used in order to identify differences in sialometry values within each group at the two study time points. With the purpose of comparing possible sialometry alterations between groups, at N0 and N30, the Mann-Whitney test was used. Values of *p* ≤ 0.05 were considered statistically significant.

We used the statistical analysis program Statistical Package for the Social Sciences, version 21.0.

## RESULTS

Demographic and clinical characteristics of the patients are shown in Table 1. Table 2 shows variables related to the tumor and its treatment.

**Table 1:** Demographic and clinical characteristics of the patients (*n* = 23).

Characteristics		Laser			Control			Total	
		N	%		N	%		N	%
Gender	Male	11	84.6		9	90.0		20	87.0
	Female	2	15.4		1	10.0		3	13.0
Ethnicity	Leukoderma	8	61.5		7	70.0		15	65.2
	Pheoderma	3	23.1		3	30.0		6	26.1
	Melanoderma	2	15.4		0	0.0		2	8.7
Alcoholism	Current	8	61.5		7	70.0		15	65.2
	Previous	3	23.1		3	30.0		6	26.1
	None	2	15.4		0	0.0		2	8.7
Smoking	Previous	10	76.9		7	70.0		17	73.9
	Current	2	15,4		2	20.0		4	17.4
	None	1	7.7		1	10.0		2	8.7

**Table 2:** Characteristics of the tumors and the treatments given.

Characteristics of tumor and treatment		Laser			Control			Total	
		N	%		N	%		N	%
Histological type	Squamous cell carcinoma	13	100.0		10	100.0		23	100.0
Anatomical site	Pharynx	8	61.5		8	80.0		16	69.6
	Larynx	2	15.4		2	20.0		4	17.4
	Occult primary tumor	2	15.4		0	0.0		2	8.7
	Oral cavity	1	7.7		0	0.0		1	4.3
Tumor stage	IV	10	76.9		9	90.0		19	82.6
	III	2	15.4		0	0.0		2	8.7
	I	1	7.7		1	10.0		2	8.7
Previous surgical treatment	No	9	69.2		6	60.0		15	65.2
	Yes	4	30.8		4	40.0		8	34.8
Total radiation dose	70 Gy	11	84.6		7	70.0		18	78.3
	66 Gy	2	15.4		3	30.0		5	21.7

**Figure 1:**
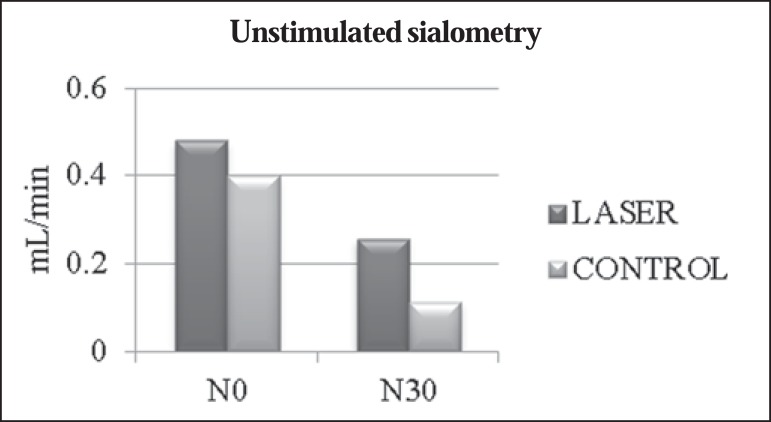
Comparisons between mean value of unstimulated salivary flow rate between patients receiving laser therapy (laser group) and patients who received only clinical care (control group) at the beginning of radiotherapy and chemotherapy (N0) and 30 days after completion (N30).

**Figure 2:**
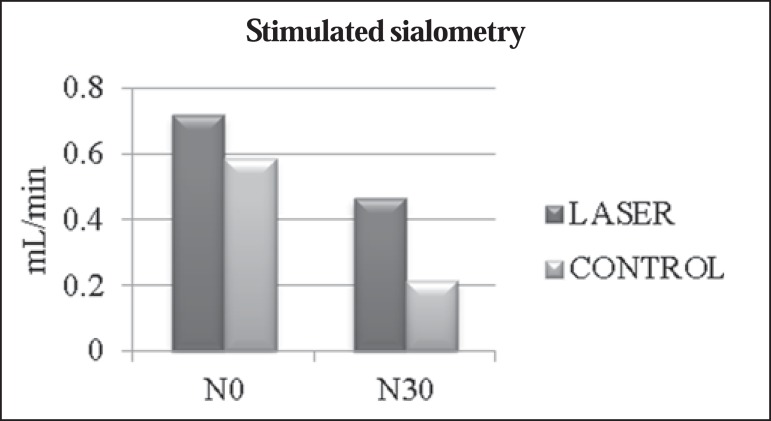
Comparisons between mean value of stimulated salivary flow rate between patients receiving laser therapy (laser group) and patients who received only clinical care (control group) at the beginning of radiotherapy and chemotherapy (N0) and 30 days after completion (N30).

The mean values obtained in the unstimulated sialometry tests are shown in Table 3 and Figure 1. Table 4 and Figure 2 show the mean values obtained in the stimulated sialometry tests.

**Table 3:** Unstimulated sialometry test values.

Mean salivary flow rate		Laser	Control	*p*-value
Unstimulated sialometry	N0	0.480	0.400	0.081
	N30	0.257	0.113	0.0143*
*p*-value		0.0096*	0.0051*	

Comparisons of mean values for unstimulated salivary flow rate between patients receiving laser therapy (laser group) and patients receiving only clinical care (control group) at the beginning of radiotherapy/chemotherapy sessions (N0) and at 30 days after their completion (N30).

* Statistically significant

**Table 4:** Stimulated sialometry test values.

Mean salivary flow rate		Laser	Control	*p*-value
Stimulated sialometry	N0	0.717	0.587	0.0771
	N30	0.463	0.213	0.0131*
*p*-value		0.0360*	0.0051*	

Comparisons of mean values for unstimulated salivary flow rate between patients receiving laser therapy (laser group) and patients receiving only clinical care (control group) at the beginning of radiotherapy/chemotherapy sessions (N0) and at 30 days after their completion (N30).

* Statistically significant.

## DISCUSSION

Saliva plays a fundamental role in the maintaining the physiological and microbiological balance in the oral cavity, as well as participating in the initial digestive processes^(7,10)^. Low salivary flow rate resulting from glandular damage is an important and common sequela in patients with HNC undergoing radiotherapy and chemotherapy^(19)^. The decreased salivary flow rate can impede basic oral functions and increase the risk of caries, periodontal disease, and opportunistic infections, directly influencing patient quality of life^(8)^. Therefore, better explanations regarding the mechanisms and processes involved in the glandular response to radiotherapy and chemotherapy are appropriate and necessary in order to establish preventive measures and effective treatments.

In the present study, at baseline (N0), the failure to obtain statistical significance between the groups of both sialometry tests underscores the initial homogeneity envisioned by the researchers, in which the eight patients who had undergone surgery were equally and randomly allocated to receive laser therapy (*n* = 4) or routine clinical care only (*n* = 4). In addition, all eight of those patients had only one submandibular gland removed. As for chemotherapy, all 23 subjects received cisplatin. The lack of information about the influence of chemotherapeutic agents on glandular functions, due to the lack of studies with standardized methods, representative samples, satisfactory duration, and dissociation of radiotherapy^(10)^, justified the inclusion of patients with similar chemotherapy regimens.

Sialometry evaluations were performed at N0 and N30 for different reasons. Given that markedly diminished salivary flow rate and high salivary viscosity are expected during cancer treatment^(9)^, the volumetric results might be inaccurate because of the influence of difficulties in the collection procedures. In this critical period, patients are also subjected to high levels of stress and generally exhibit inflammatory reactions in the mucosa (mucositis). The use of 2% sodium citrate as a gustatory stimulant could exacerbate irritation of the mucosa and increase pain levels.

Our results show a significant reduction in mean salivary flow rate in both sialometry tests and in both study groups. Lopes et al.^(20)^, who carried out a study with objectives and methodology similar to those of the present study, albeit with different laser therapy parameters, also reported progressive drops in the mean values obtained in the sialometry tests in the group not subjected to laser treatment, at different post-radiotherapy time points, including 30 days later.

Regarding unstimulated salivary flow rate, we observed that patients who received laser therapy showed a reduction of approximately 0.223 mL/min (46.5%), compared with 0.287 mL/min (71.75%) for those who did not. Extrapolating these data, we can corroborate those in the literature, which states that, for radiotherapy patients (submitted to surgery or not) in whom no preventive measures are taken, unstimulated salivary flow rate can decrease by up to 45% of the initial value during radiotherapy and continue to progressively decrease until the end of radiotherapy^(7)^. In fact, in our patients undergoing laser therapy, some degree of reduction due to radiotherapy and chemotherapy was also expected. However, it occurred with less intensity, underscoring the benefits of laser therapy in preventing this side effect. According to the unstimulated salivary flow rate scale proposed by Eisbruch et al.^(17)^, the patients in our laser group showed no hyposalivation or mild hyposalivation (mean salivary flow rates > 0.2 mL/min) at N30, whereas those in the control group showed moderate hyposalivation (mean salivary flow rates of 0.1-0.2 mL/min) at the same time point.

Regarding stimulated salivary flow rate, the laser group showed a mean decrease of 0.254 mL/min (35.4%), compared with 0.374 mL/min (63.7%) for the control group. The literature indicates that patients subjected only to radiotherapy and with no additional preventive treatment for low salivary flow rate demonstrate reductions in the mean stimulated sialometry values^(2,18)^ of approximately 64% immediately after radiotherapy (approximately 70 Gy) and of 74% after two months^(12)^. To our knowledge, there have been no studies employing a methodology similar to ours in order to investigate stimulated sialometry values at 30 days after the end of radiotherapy and chemotherapy. In our study, we found that laser therapy was beneficial in preventing a reduction not only in stimulated salivary flow rate but also in unstimulated salivary flow rate.

The use of our intraoral laser therapy protocol, which covered sublingual glands and other minor salivary glands distributed throughout the oral cavity, in combination with our extraoral protocol for applying laser therapy in order to stimulate the parotid and submandibular glands directly, was conceptualized based on reports of increased salivary flow rates and decreased xerostomia when laser therapy is used for mucositis in patients irradiated for HNC^(3,20,26,29)^. To our knowledge, there have been no previous studies employing laser therapy protocols specifically designed for direct stimulation of the salivary glands in HNC patients and combining different wavelengths in extraoral and intraoral applications.

Considering the limitations of our study, we believe that the results were satisfactory. The maintenance of salivary flow rate at 30 days after the last session of radiotherapy and chemotherapy was clearly more common in patients who received laser therapy during the course of treatment. Although the laser therapy protocol required a large number of application points and was performed for a relatively long period, it was well tolerated by patients, who were more responsive to treatment. In addition, it proved feasible in a public hospital that receives a large number of patients on a daily basis.

## CONCLUSION

Evaluated together, our results show that low-level laser therapy is an effective agent for the attenuation of low salivary flow rates after radiotherapy and chemotherapy. The maintenance of salivary flow within the normal range after the completion of cancer treatment is quite desirable, allowing other potential and late radiotherapy- and chemotherapy-induced effects to be prevented or mitigated, as well as ensuring that such treatment will have less of an impact on the quality of life of the affected patients.
